# Changes of Soil Particle Size Distribution in Tidal Flats in the Yellow River Delta

**DOI:** 10.1371/journal.pone.0121368

**Published:** 2015-03-27

**Authors:** Xiaofei Lyu, Junbao Yu, Mo Zhou, Bin Ma, Guangmei Wang, Chao Zhan, Guangxuan Han, Bo Guan, Huifeng Wu, Yunzhao Li, De Wang

**Affiliations:** 1 Key Laboratory of Coastal Environmental Processes and Ecological Remediation, Yantai Institute of Coastal Zone Research (YIC), Chinese Academy of Sciences (CAS); Shandong Provincial Key Laboratory of Coastal Environmental Processes, YICCAS, Yantai, Shandong, China; 2 University of Chinese Academy of Sciences, Beijing, Beijing, China; 3 Environment College, Northeast Normal University, Changchun, 130024, P. R. China; Centro de Investigacion Cientifica y Educacion Superior de Ensenada, MEXICO

## Abstract

**Background:**

The tidal flat is one of the important components of coastal wetland systems in the Yellow River Delta (YRD). It can stabilize shorelines and protect coastal biodiversity. The erosion risk in tidal flats in coastal wetlands was seldom been studied. Characterizing changes of soil particle size distribution (PSD) is an important way to quantity soil erosion in tidal flats.

**Method/Principal findings:**

Based on the fractal scale theory and network analysis, we determined the fractal characterizations (singular fractal dimension and multifractal dimension) soil PSD in a successional series of tidal flats in a coastal wetland in the YRD in eastern China. The results showed that the major soil texture was from silt loam to sandy loam. The values of fractal dimensions, ranging from 2.35 to 2.55, decreased from the low tidal flat to the high tidal flat. We also found that the percent of particles with size ranging between 0.4 and 126 μm was related with fractal dimensions. Tide played a great effort on soil PSD than vegetation by increasing soil organic matter (SOM) content and salinity in the coastal wetland in the YRD.

**Conclusions/Significance:**

Tidal flats in coastal wetlands in the YRD, especially low tidal flats, are facing the risk of soil erosion. This study will be essential to provide a firm basis for the coast erosion control and assessment, as well as wetland ecosystem restoration.

## Introduction

Tidal flat is the tide-dominant coastal wetland. It can provide ecosystem services linked to nutrient uptake and retention [1]. Tidal flats supply organic matters to adjacent rivers and coastal zones, and support high trophic levels in a range of aquatic habitats [2,3]. Moreover, it plays an important role in preventing erosion of coastal lines and seawater contamination.

Tidal flats are generally found several hundreds of meters wide along the coast with varying vegetation types and soil properties [4]. Tidal flats are immature, fragile and unstable [5]. Tidal currents have been identified as one of the dominant factor controlling coastal wetland ecosystem evolution [6–8]. Tidal flat had led to great understanding of their place in ecosystem; however, it has not been fully explored yet. More information is needed regarding details of the relationship of natural disturbances and soil properties on tidal flats. Especially, little is known about an effective index to quantify the effects on soil properties affected by tidal action.

Soil particle size distribution (PSD), one of the most important physical attributes of soils, may change correspondingly in different soil conditions. Characterizing changes of soil PSD is an important way to understand and quantify soil structure, dynamics and physical process [9–14]. The traditional and principal approach for soil PSD, the textural triangle, provides incomplete information for its frequently fluctuations and restriction by arbitrary of texture classes [15]. A better approach to characterize PSD is combining laser diffraction method and fractal analysis, which offers the possibility for quantifying and integrating information on soil structure at different temporal and spatial scales [14,16–19]. Singular fractal analysis is used to quantitatively describe soil PSD characteristics, soil aggregate fragmentation, and other related soil properties [20]. Multifractal analysis has been employed to retain more detailed information to capture the intrinsic variability of soil PSD [11,14,19,21].

In this study, we calculated both singular and multiple fractal dimensions of soil PSD with fractal scale theory, and explored the relationships between soil particle sizes and fraction dimensions with network analysis in a successional series of tidal flats in a coastal wetland in the YRD. The objectives of this work were to: 1) analyze the soil PSD and its fractal dimensions in tidal flat in the YRD, and 2) explore the related soil properties of soil PSD in tidal flats.

## Methods

### Ethics statement

Our study area is located in the Yellow River Delta Natural Reserves, which is owned by the Chinese government. We obtained a specific permit from the administration bureau of the Yellow River Delta National Nature Reserve for conducting research in the preserve. Moreover, our sampling sites were not located in any strictly protected areas containing endangered or protected species.

### Study area

The Yellow River Delta (YRD) (118.6^o^E-119.3°E, 37.6^o^N-38.2^o^N), located in northern part of Shandong Province, has the largest and youngest coastal wetlands in China. It is one of the most active regions of land-ocean interaction among the large river deltas in the world. The region is a warm-temperature and semi-humid continental monsoon climate, with annual average temperature of 11.7–12.6°C. The annual evaporation is 1900–2400 mm and annual precipitation is 530–630 mm, of which 70% is rainfall during June to August.

**Fig 1:**
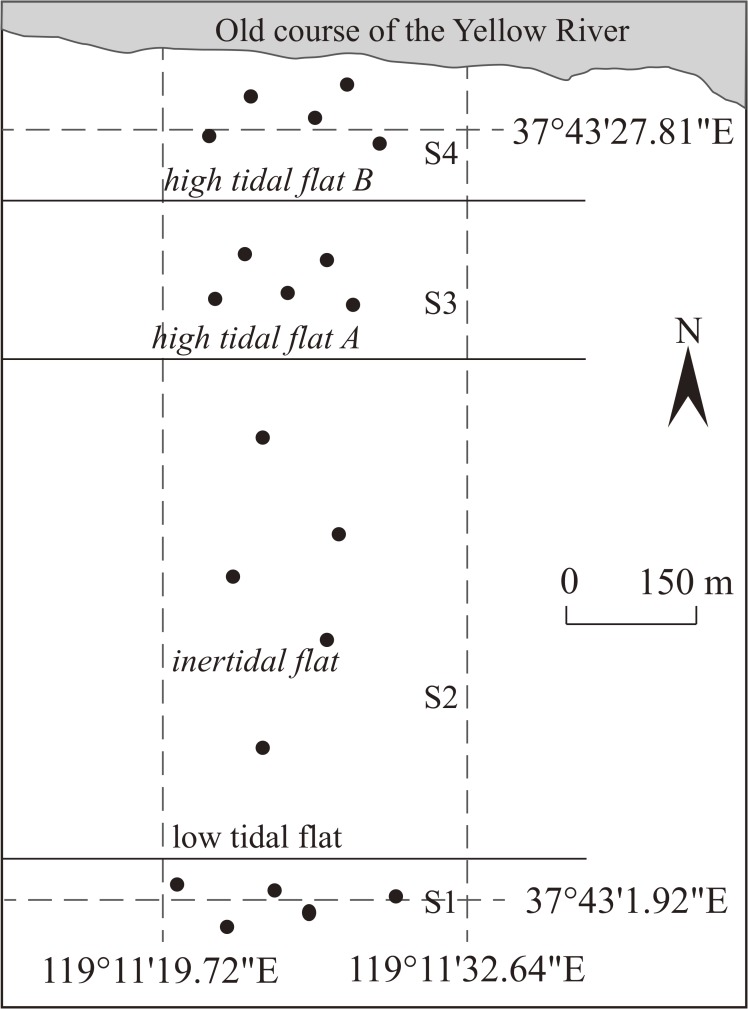
The location of study region and sampling sites.

Tidal flats in the YRD have clear horizontal distribution vegetation zones from low tidal flats to high tidal flats. Along the seaside to the inland, four sites located in the low tidal flat (S1), the intertidal flat (S2), the high tidal flat A (S3), and the high tidal flat B (S4), respectively, were selected. Location and detail information of each tidal flat are provided in Fig. 1 and Table 1, respectively. No plant can grow in S1 plot as soil salinization progresses, and *Tamarix chinesis* in S2 are threatened. High tidal flats S3 and S4, which are seldom affected by tidal actions, are undergoing a natural succession process with different vegetation.

**Table 1:** General condition of the sampling sites.

Sites	S1	S2	S3	S4
Main vegetation community	None	*Tamarix chinesis*	*Phragmites australis*	*Imperata cylindrica*
Coverage of plant (%)	0	35	100	100
Elevation (m)	2.2	2.6	3.2	3.5
Distance to the low tidal line (m)	0	200	670	850

S1-Low tidal flat; S2-Intertidal flat; S3-High tidal flat A; S4-High tidal flat B.

### Sampling and processing

Soil samples were collected from four plots during July 2012 to May 2013. After carefully removing surface organic materials and fine roots, soil samples were collected at three soil depths: 0–10 cm, 10–20 cm and 20–30 cm. The five replicate samples were homogenized mixed. Visible roots and rocks were removed prior to further processing.

All the air-dried soil samples were dispersed by sodium hexametaphosphate (NaHMP) and ultrasonic lasting 30 s after manually sieved (2 mm) and homogenized. The particle fractions of samples were analyzed with Longbench Mastersizer 2000 (Malvern Instruments, Malvern, England). Soil salinity was quantified as soil electrical conductivity (EC, μS cm^− 1^) by measuring the EC of suspension with a 1:5 soil water ratio after 1 h suspension shaking at 25°C. Soil pH was measured using a glass electrode. Soil organic matter (SOM) content was determined by K_2_CrO_7_ routine colorimetric method [22].

### Data analyses

In the study, soil PSD ranging from 0.2 μm to 2000 μm was obtained representing relative volume (%) versus soil particle diameter (μm). The interval of particle sizes (μm) *I* = [0.2, 2000] were graded into 64 subintervals *I*_*i*_ = [Ø_*i*_, Ø_*i+*1_], *i* = 1, 2, …, 64, and the lengths of subintervals follows a logarithmic scale such that log(Ø_*i+*1_/ Ø_*i*_) was constant. Meanwhile, according to the United States Department of Agriculture classification of soil particle size, the soil size was partitioned into 3 grades, clay (0–2 μm), silt (2–50 μm) and sand (50–2000 μm).

The singular fractal dimension (*D*) of soil PSD was estimated from the following equation [17]:

$$\frac{V(r<R_{i})}{V_{T}}={\left(\frac{R_{i}}{R_{max}}\right)}^{3-D}$$ (1)

Where *V*_(*r <Ri*)_ is the cumulative percentage of particles of *i* size *r* less than *R*_*i*_, *V*_*T*_ is the total percentage (*V*_*T*_ = 100), *R*_*i*_ is the particle radius (mm) of the *i*th size class, and *R*_*max*_ is the radius of the largest particle class (*R*_*max*_ = 1, in this study). The particle diameter is taken as the upper sieve sizes. Taking logarithms on both sides of Eq. (1), the *D* value can be derived by the slopes of the logarithmic linear regression equation.

The multifractal dimension (Rényi dimension) was also used. A number of cells with size of *ε* to cover the entire interval, and the cell number is *N*, the Rényi dimension is computed by the mass of soil particles in subinterval, *μ_i_*(*ε*) cell diameter *ε* and the parameter *q* [16,23]. *D*_*q*_ extracts the system parameters from different levels with the *q* value in the interval [∞, +∞].

$$D_{1}=\lim_{\varepsilon \to 0}\frac{\sum_{i=1}^{N(\varepsilon )}\mu_{i}(\varepsilon )\log\mu_{i}(\varepsilon )}{\log(\varepsilon )}\;\left(q=1\right)$$ (2)

$$D_{q}=\frac{1}{q-1}\lim_{\varepsilon \to 0}\frac{{\log\sum }_{i=1}^{N(\varepsilon )}\mu_{i}{\left(\varepsilon \right)}^{q}}{\log(\varepsilon )}\;\left(q\neq 1\right)$$ (3)

When q = 0, *D*_0_ is the capacity dimension known as box-counting dimension. It reflects the range of a continuous distribution. *D*_0_ = 1 means that the interval of particle-size from 0.2 to 2000 μm are all occupied at all scales. When q = 1, the entropy dimension *D*_1_ provides a measure of the heterogeneity of soil PSD [19]. High value of *D*_1_ means high degree of heterogeneous of soil’s PSD. Considering that *D*_0_ provides general information and *D*_1_ measures the homogeneity of PSD system, *D*_1_/*D*_0_ is used to quantify the dispersion of the measurement over the set of sizes to obtain the relation between the two parameters. As *D*_1_ takes value less than *D*_0_, the quotient *D*_1_/*D*_0_ is less than 1. The closer to 1 *D*_1_/*D*_0_ is, the more evenly dispersed is the fractions over the set of sizes, the more heterogeneous in the distribution. Consequently, the particle of samples disperses over the set of sizes for the relative high values of *D*_1_/*D*_0_.

Data in the figures and tables were mean values of each sample. All statistical analyses were implemented using various packages within the R statistical computing environment. One-way analysis of variance (ANOVA) procedures were used to detect differences in measured parameters among soil samples. Network analyses were used to show the composition of, and interactions between, multiple elements in communities. A matrix of correlation between all trait pairs was generated by network analysis. Significance levels were set at *p* < 0.05. The total significant pairs resulted were considered as a network in which a vertex corresponds to a trait and a link between two vertices corresponds to significant correlations between these two traits. This network plot was then subjected based on the adjacency matrix with *igraph* package.

## Results

### Soil particle size distribution

**Fig 2:**
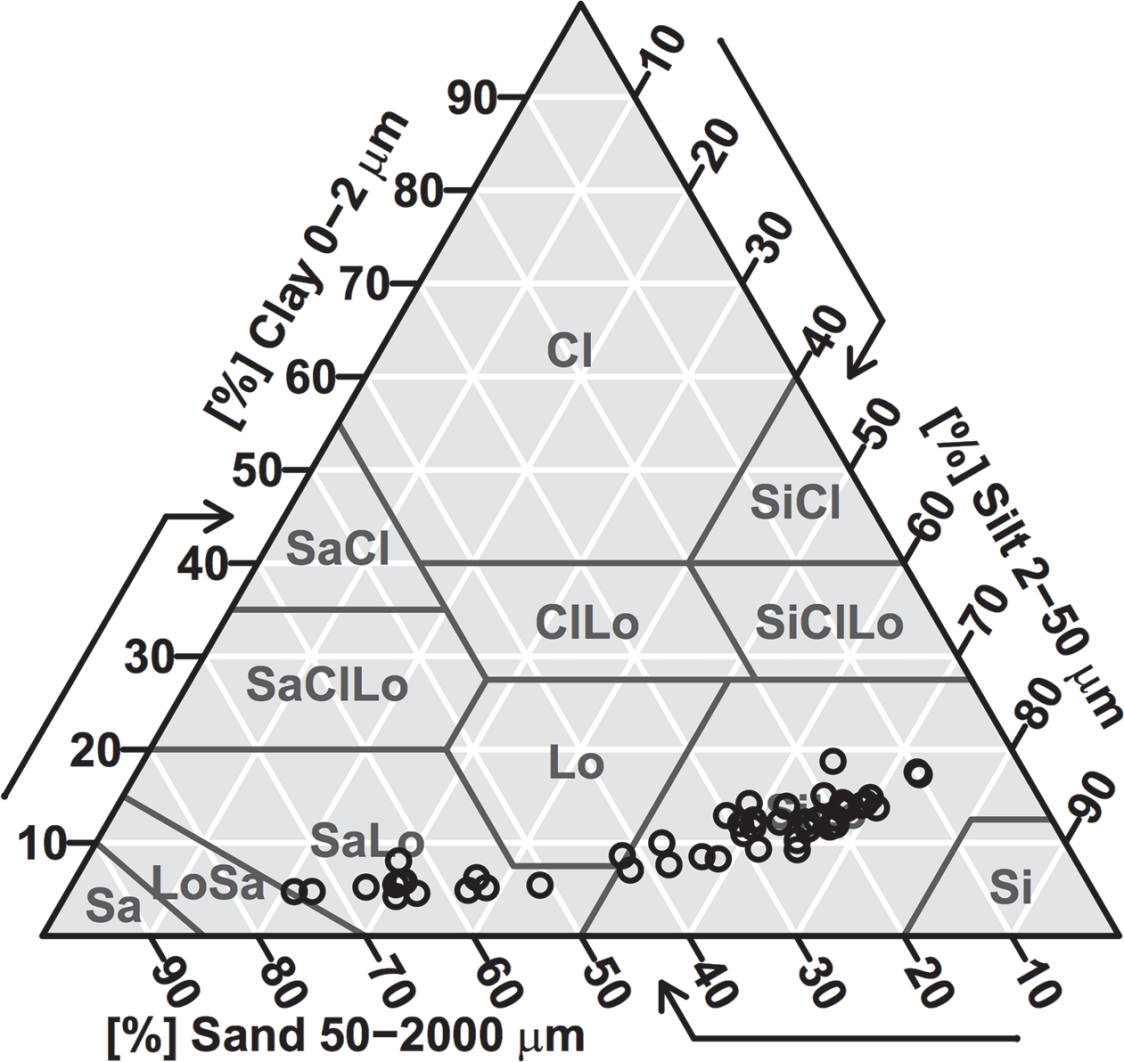
Texture of analyzed soil samples.

**Fig 3:**
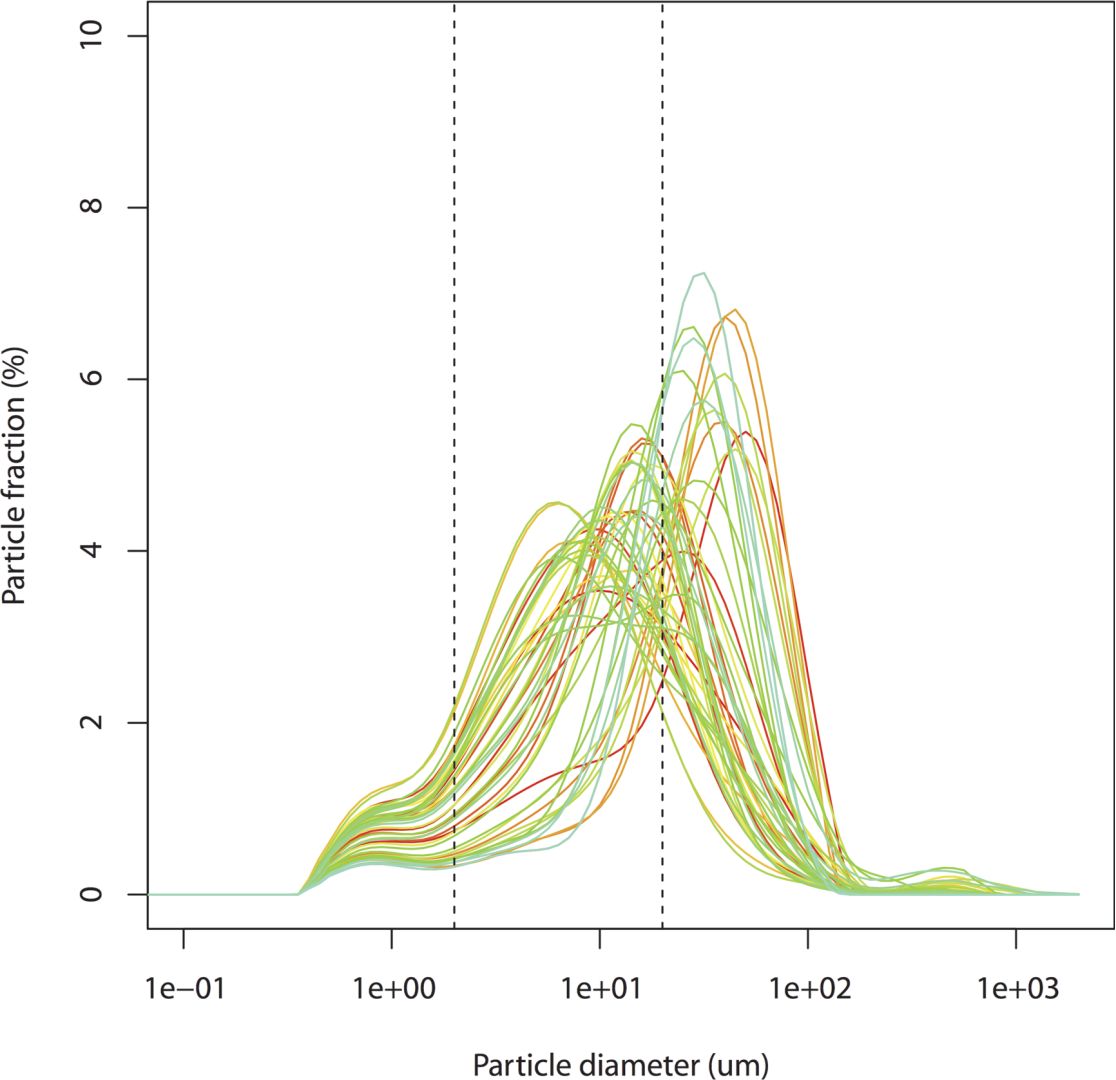
Relatively percentages of particle size volumes in the 48 soil samples.

The major soil texture was from silt loam to sandy loam in present study (Fig. 2). The soil texture in soils was classified as silt loam in the low tidal flat (S1), the intertidal flat (S2), and the high tidal flat A (S3). Silt particles were the predominant soil particles, with a mean value of 62% (Fig. 3, Table 2). Sand and clay represented a mean value of 26% and 12%, respectively. The soil texture in soils can be classified as sandy loam in high tidal flat (S4). Sand, silt and clay fraction contents were 63%, 32% and 5%, respectively. The PSD was statistically significant different among tidal flats. The fine particle (clay and silt) content was the highest in February, and the lowest in July.

**Table 2:** Fractal dimensions (*D*, *D*_0_, *D*_1_, and *D*_1_/*D*_0_), soil texture, and soil properties in different factors.

Factor	Variation	*D*	*D*_0_	*D*_1_	*D*_1_/*D*_0_	Clay	Silt	Sand	pH	Salt (g kg^−1^)	SOM (%)
Tidal flats	S1	2.53	0.89	0.81	0.91	13.78	60.20	26.02	8.30	2.38	1.24
	S2	2.52	0.90	0.80	0.90	12.93	65.26	21.81	8.15	1.91	1.63
	S3	2.48	0.90	0.79	0.88	9.88	61.21	28.80	8.14	0.43	1.55
	S4	2.38	0.89	0.76	0.86	5.21	31.83	62.96	8.09	0.33	1.04
	F values	23.0	0.27	23.0	15.12	48.88	56.67	55.14	2.92	41.75	8.27
	*p* values	<0.001	0.85	<0.001	<0.001	<0.001	<0.001	<0.001	0.05	<0.001	<0.001
Soil depth	0–10	2.48	0.90	0.80	0.89	10.06	53.11	36.83	8.15	1.26	1.88
	10–20	2.49	0.90	0.79	0.88	10.97	57.90	31.13	8.18	1.20	1.25
	20–30	2.48	0.88	0.78	0.89	10.32	52.94	36.74	8.20	1.33	0.96
	F values	0.40	2.93	0.40	2.51	0.46	2.53	2.19	0.25	0.24	33.26
	*p* values	0.76	0.07	<0.001	0.09	0.71	0.09	0.14	0.78	0.79	<0.001
Seasonal variation	July (2012)	2.48	0.89	0.79	0.89	10.14	49.81	40.04	8.12	1.46	1.23
	Oct (2012)	2.49	0.90	0.80	0.88	10.97	56.19	32.84	8.58	1.53	1.72
	Feb (2013)	2.48	0.89	0.79	0.88	10.13	57.48	32.39	7.66	1.00	1.41
	May (2013)	2.48	0.89	0.79	0.88	10.55	55.12	34.33	7.63	1.07	1.09
	F values	0.62	0.52	9.71	0.13	0.46	2.71	1.92	64.35	2.76	8.25
	*p* values	0.61	0.67	0.76	0.93	0.71	0.06	0.14	<0.001	0.06	<0.001

### Fractal dimension characteristics of soil PSD

The singular fractal dimension (*D*) values for PSD, calculated using Eq. (1), ranged from 2.35 to 2.55 in all soil samples. The *D* values were statistically different among four tidal flats (*p* < 0.01) (Table 2), followed the trend of S1 > S2 > S3> S4.

**Fig 4:**
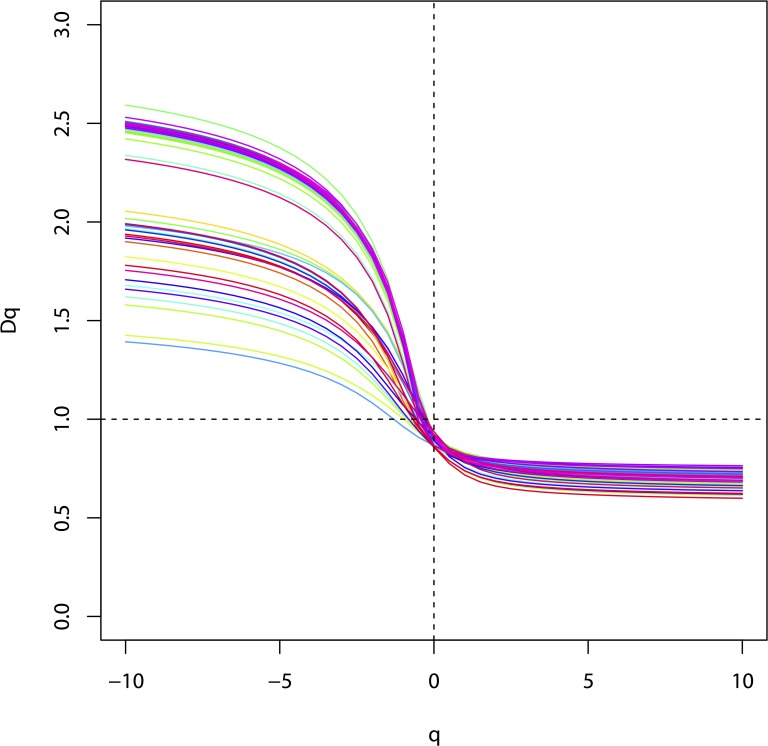
The Rényi dimensions spectra D_q_-q curves of soil samples.

The multifractal dimension *D*_1_ and *D*_0_ were calculated via Eq. (2) and Eq. (3), respectively. The entropy dimension (*D*_1_) achieved values from 0.71 to 0.83. The capacity dimension (*D*_0_) varied from 0.85 to 0.94. (Fig. 4, Table 2). The values of *D*_1_/*D*_0_ ranged from 0.83 to 0.94. According to analysis of variance, values of *D*_1_/*D*_0_ had a statistically significant difference (*p* < 0.01) among tidal flats. Values of *D*_1_ had significant differences among tidal flats (*p* < 0.01) and soil depth variance (*p* < 0.01).

### Relationship between soil particle size distribution and soil properties

**Fig 5:**
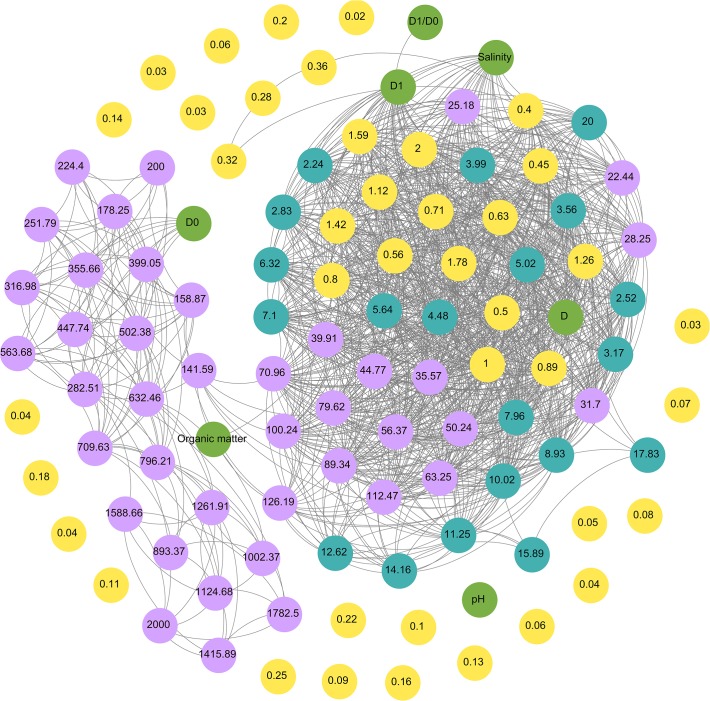
Network plot shows the associations between soil texture, fractal parameters and soil properties. The lines indicate significant correlations (*p* < 0.05). The number in the dots refers to the size of soil particles.

We found the similar tendency towards soil fractal parameters and physicochemical properties (Table 1). A correlation network was built to test co-occurrence pattern between soil particle size distribution and soil physicochemical property (Fig. 5). All significant correlations (*p* < 0.05) were visualized as edges in the network.

The resulting network contained two modules: one was large and densest connected, the other one was small and weak connected. The large and interconnected module contained content of soil particle with size ranging between 0.40 μm and 126 μm. The nodes for soil salinity, *D* values, and *D*_1_ values were also included in this module. The small loosely interconnected modules contained *D*_0_ and a set of 126–2000 μm soil particle size content. The nodes represented *D*_1_, connecting the largest module and two nodes: SOM content and *D*_1_/*D*_0_.

## Discussion

### Fractal property features in the YRD

Comparing with other regions under different land-use in the YRD, the *D* values in tidal flats (2.35∼2.55) were similar to those in grass and farm (2.46∼2.60), less than those in forest (2.50∼2.68) [14]. Values of *D* have a positive correlation with fine fraction content [24,25]. Several studies show that *D* values in soils with fine texture typically range from 2.60 to 2.80, and soils with poor structure and coarse texture range from 1.80 to 2.60 [13,18,26]. Accordingly, the soil structure in the tidal flats in the coastal wetland in the YRD was relatively poor.

Values of *D*_0_ were stable in different tidal flats, whereas the values of *D*_1_ and *D*_1_/*D*_0_ decreased from the low tidal flat to the high tidal flat. The value of *D*_0_ reflects the range of the soil PSD; the values of *D*_1_ and *D*_1_/ *D*_0_ describe the degree of disorder and heterogeneity of the soil PSD [19]. Therefore, the degree of disorder and heterogonous was declined from the low tidal flat to the high tidal flat. The scouring effects of tidal action could explain these results. These effects decrease from the low tidal flat to the high tidal flat. Thus, the sand content increased and silt content decreased from the low tidal flat to the high tidal flat. These traits result in the greater irregularity and heterogeneity of the soil PSD in the low tidal flat and further verify the order that singular fractal dimension in tidal flats in the YRD.

### Related soil properties

Soil PSD closely interacts with their surrounding environment, and the interaction changes with the development of wetlands [8,24]. The SOM and salinity were the factors related with fractal dimension of PSD in tidal flats in coastal wetlands in the YRD. The organic carbon accumulation in soil can improve soil structure, and increase soil micro-aggregates and fine particle fractions corresponding to the fractal dimension of PSD [8,24]. Salinity has a pronounced negative effect on SOM decomposition, soil respiration, and microbial activity, increasing fine fragment content irrespective of soil texture [27,28]. The concentration of the aromatic dissolved organic carbon fraction could increase to protect clay minerals against degradation in salt-affected landscapes [27,29].

Tidal action alters the soil PSD by increasing SOM content and salinity in coastal wetland in the YRD. The majority of SOM comes from tidal actions, which could shift algae, marine animals, and organic materials to tidal flats in coastal wetlands [6,30]. The SOM content in the low tidal flat is less than those in other flats, because the soil dissolved organic matter can dissolve under tidal hydraulic action [31]. Saline water intrusions could increase the soil salinity in coastal wetland, especially in low tidal flats and intertidal flats [32].

This rank series of fractal dimension was opposed to those in the coastal wetland of Chongming and mangrove forest [33,34], where the fine fraction increased from subtidal flat to supratidal flat. Previous studies showed soil has a high *D* value with flush vegetation [7,11,21]. Vegetation coverage has a major impact on increasing fine particles content and nutrients and decreasing the risk of soil erosion [7,21]. However, plant productivity is relatively low in coastal wetland in the YRD because this landscape was formed in less than 40 years (since1976). Once the influence of freshwater from Yellow River increases and tidal energy from ocean decreases, plant productivity and fractal dimensions would increase in high tidal flat. It indicates that the tidal action on fining soil texture is greater than the plants effort in coastal wetland in the YRD.

### The various particle sizes related with different fractal dimension

Using Network analysis, we found that the values of *D*, *D*_1,_ and D_1_/ *D*_0_ in coastal wetland had strong positive relationship with content of fine particles (0.40–126 μm), and *D*_0_ was reasonably correlated with content of coarse particles (126–2000 μm). Previous studies also show that *D* values increase with the rise of fine particles content, especially clay content [13,21,26]. Values of *D*_1_ and *D*_1_/ *D*_0_ are also significantly positively correlated with fine particle content in different landscapes under different conditions [13]. These indicate that distribution heterogeneity increases with fine particle content. The smaller particle sizes in the soil mean the greater spatial-filling capacity of the soil, corresponding to the higher fractal dimension values in the soil [26]. Usually, we pay more attention on fine particles because they have strong relationship with soil quality. Moreover, fine particles could provide adequate details to identify associations and differences between soil samples [35]. The fractal dimension *D*_0_ can provide useful information about coarse particles, which is ignored by other fractal dimensions. Here, we firstly reported that the size of fine particles that content was related with values of fractal dimensions (*D*, *D*_1_, and *D*_1_/ *D*_0_) ranged between 0.4 and 126 μm.

The soil with more fine particles may be more susceptible to erosion [36]. The soils in low tidal flats and intertidal flats are eroded by tidal action. The loss of fine particles in the coastal wetland would induce the fractal dimension values declination, and subsequently enhance the risk of coastline erosion, wetlands degradation, and soil salinization.

Soil PSD is closely related with soil functions and they are interdependent [14]. Fractal parameters have been considered as potential indicators to reflect the effects of environments variations on soil texture, soil aggregate, and other soil properties [9,11,20]. It also can be considered as a sensitive and practical index for quantifying changes evaluating wetland degradation and coastal erosion.

## Conclusions

In the present study, the fractal dimension values decreased along the low tidal flat to the high tidal flat in coastal wetland in the YRD, indicating different erosion degree in tidal flats. Our results suggest that the tide plays more important role in tidal flats in the YRD. Based on network analysis, we firstly reported that the size of soil particles that was related with fractal dimensions was between 0.4 and 126 μm. Our results underscore the importance of fractal dimension when assessing the coastal erosion and wetland degradation in the YRD.

## References

[1] Fan XM, Pedroli B, Liu GH, Liu HG, Song CY, Shu LC. Potential plant species distribution in the Yellow River Delta under the influence of groundwater level and soil salinity. Ecohydrology. 2011;4(6):744–756.

[2] Baker R, Fry B, Rozas LP, Minello TJ. Hydrodynamic regulation of salt marsh contributions to aquatic food webs. Mar Ecol Prog Ser. 2013;490:37–52.

[3] Doi H. Spatial patterns of autochthonous and allochthonous resources in aquatic food webs. Popul Ecol. 2009;51(1):57–64.

[4] Schile LM, Callaway JC, Morris JT, Stralberg D, Parker VT, Kelly M. Modeling tidal marsh distribution with sea-level rise: Evaluating the role of vegetation, sediment, and upland habitat in marsh resiliency. Plos One. 2014;9(2):e88760 10.1371/journal.pone.0088760 24551156 PMC3923833

[5] Yu J, Fu Y, Li Y, Han G, Wang Y, Zhou D, et al Effects of water discharge and sediment load on evolution of modern Yellow River Delta, China, over the period from 1976 to 2009. Biogeosciences. 2011;8(9):2427–2435.

[6] Mariotti G, Fagherazzi S. Critical width of tidal flats triggers marsh collapse in the absence of sea-level rise. Proc Natl Acad Sci USA. 2013;110(14):5353–5356. 10.1073/pnas.1219600110 23513219 PMC3619298

[7] Zhang P, Wei T, Jia Z, Han Q, Ren X, Li Y. Effects of Straw Incorporation on Soil Organic Matter and Soil Water-Stable Aggregates Content in Semiarid Regions of Northwest China. Plos One. 2014;9(3):e92839 10.1371/journal.pone.0092839 24663096 PMC3963976

[8] Wissing L, Kölbl A, Schad P, Bräuer T, Cao Z-H, Kögel-Knabner I. Organic carbon accumulation on soil mineral surfaces in paddy soils derived from tidal wetlands. Geoderma. 2014;228:90–103.

[9] Salako FK, Babalola O, Hauser S, Kang BT. Soil macroaggregate stability under different fallow management systems and cropping intensities in southwestern Nigeria. Geoderma. 1999;91(1):103–123.

[10] Gulser C. Effect of forage cropping treatments on soil structure and relationships with fractal dimensions. Geoderma. 2006;131(1):33–44.

[11] Martin MA, Garcia-Gutierrez C, Reyes M. Modeling Multifractal Features of Soil Particle Size Distributions with Kolmogorov Fragmentation Algorithms. Vadose Zone J. 2009;8(1):202–208.

[12] Ghorbani N, Raiesi F, Ghorbani S. Bulk soil and particle size-associated C and N under grazed and ungrazed regimes in Mountainous arid and semi-arid rangelands. Nutr Cycl Agroecosys. 2012;93(1):15–34.

[13] Xu GC, Li ZB, Li P. Fractal features of soil particle-size distribution and total soil nitrogen distribution in a typical watershed in the source area of the middle Dan River, China. Catena. 2013;101:17–23.

[14] Peng G, Xiang N, Lv S-q, Zhang G-c. Fractal characterization of soil particle-size distribution under different land-use patterns in the Yellow River Delta Wetland in China. J Soil Sediment. 2014;14(6):1116–1122.

[15] Montero E, Martin MI. Holder spectrum of dry grain volume-size distributions in soil. Geoderma. 2003;112(3):197–204.

[16] Billingsley P. Ergodic theory and information: Wiley New York; 1965.

[17] Tyler SW, Wheatcraft SW. Fractal Scaling of Soil Particle-Size Distributions—Analysis and Limitations. Soil Sci Soc Am J. 1992;56(2):362–369.

[18] Su YZ, Zhao HL, Zhao WZ, Zhang TH. Fractal features of soil particle size distribution and the implication for indicating desertification. Geoderma. 2004;122(1):43–49.

[19] Wang D, Fu BJ, Zhao WW, Hu HF, Wang YF. Multifractal characteristics of soil particle size distribution under different land-use types on the Loess Plateau, China. Catena. 2008;72(1):29–36.

[20] Perfect E. Fractal models for the fragmentation of rocks and soils: a review. Eng Geol. 1997;48(3):185–198.

[21] Biswas A, Zeleke TB, Si BC. Multifractal detrended fluctuation analysis in examining scaling properties of the spatial patterns of soil water storage. Nonlinear Proc Geoph. 2012;19(2):227–238.

[22] Schulte E. Recommended soil organic matter tests. Recommended soil testing procedures for the northeastern United States Northeast Regional Bull. 1995;493:47–56.

[23] Renyi A. Probability theory. 1970. North-Holland Ser Appl Math Mech. 1970.

[24] Xie XJ, Wei FQ. Soil aggregates and fractal features under different land use types in a frequent debris flow area. J Mt Sci-Engl. 2013;10(3):437–444.

[25] Gao G-L, Ding G-D, Wu B, Zhang Y-Q, Qin S-G, Zhao Y-Y, et al Fractal scaling of particle size distribution and relationships with topsoil properties affected by biological soil crusts. Plos One. 2014;9(2):e88559 10.1371/journal.pone.0088559 24516668 PMC3917891

[26] Liu X, Zhang GC, Heathman GC, Wang YQ, Huang CH. Fractal features of soil particle-size distribution as affected by plant communities in the forested region of Mountain Yimeng, China. Geoderma. 2009;154(1):123–130.

[27] Setia R, Marschner P, Baldock J, Chittleborough D, Verma V. Relationships between carbon dioxide emission and soil properties in salt-affected landscapes. Soil Biol Biochem. 2011;43(3):667–674.

[28] Setia R, Marschner P, Baldock J, Chittleborough D, Smith P, Smith J. Salinity effects on carbon mineralization in soils of varying texture. Soil Biol Biochem. 2011;43(9):1908–1916.

[29] Mavi MS, Marschner P, Chittleborough DJ, Cox JW, Sanderman J. Salinity and sodicity affect soil respiration and dissolved organic matter dynamics differentially in soils varying in texture. Soil Biol Biochem. 2012;45:8–13.

[30] Chen M, Maie N, Parish K, Jaffe R. Spatial and temporal variability of dissolved organic matter quantity and composition in an oligotrophic subtropical coastal wetland. Biogeochemistry. 2013;115(1–3):167–183.

[31] Callaway JC, Borgnis EL, Turner RE, Milan CS. Carbon sequestration and sediment accretion in San Francisco Bay tidal wetlands. Estuaries Coasts. 2012;35(5):1163–1181.

[32] Yu J, Li Y, Han G, Zhou D, Fu Y, Guan B, et al The spatial distribution characteristics of soil salinity in coastal zone of the Yellow River Delta. Environ Earth Sci. 2014;72(2):589–599.

[33] Zhang YN, Li YL, Wang L, Tang YS, Chen JH, Hu Y, et al Soil microbiological variability under different successional stages of the Chongming Dongtan wetland and its effect on soil organic carbon storage. Ecol Eng. 2013;52:308–315.

[34] Nguyen HYT, Cao DM, Schmitt K. Soil particle-size composition and coastal erosion and accretion study in Soc Trang mangrove forests. J Coast Conserv. 2013;17(1):93–104.

[35] Pye K, Blott SJ, Croft DJ, Witton SJ. Discrimination between sediment and soil samples for forensic purposes using elemental data: an investigation of particle size effects. Forensic Sci Int. 2007;167(1):30–42. 16842947 10.1016/j.forsciint.2006.06.005

[36] Fu H, Pei SF, Wan CG, Sosebee R. Fractal Dimension of Soil Particle Size Distribution Along an Altitudinal Gradient in the Alxa Rangeland, Western Inner Mongolia. Arid Land Res Manag. 2009;23(2):137–151.

